# Multimorbidity and atrial fibrillation: Envisioning integrated approaches to complex health needs

**DOI:** 10.1016/j.ijcha.2025.101726

**Published:** 2025-06-19

**Authors:** Giulio Francesco Romiti, Gregory Y.H. Lip

**Affiliations:** a Department of Translational and Precision Medicine, Sapienza – University of Rome, Rome, Italy; b Liverpool Centre for Cardiovascular Science at University of Liverpool, Liverpool John Moores University and Liverpool Heart & Chest Hospital, Liverpool, United Kingdom; c Department of Clinical Medicine, Aalborg University, Aalborg, Denmark; d Department of Cardiology, Lipidology and Internal Medicine with Intensive Coronary Care Unit, Medical University of Bialystok, Bialystok, Poland

The epidemiology of atrial fibrillation (AF) has changed over the last decades, such that contemporary patients with AF present with more complex clinical profiles, and an overall higher burden of comorbidities, compared to 25 years ago [1]. These changes have contributed to the evolving trends of AF trajectories. Despite a general reduction in the incidence of stroke and cardiovascular mortality [1,2] (also reflecting the global increase in the uptake of oral anticoagulants), epidemiological studies showed marginal or no improvement in non-cardiovascular mortality over the last decades in patients with AF, with an increased incidence of hospitalizations for non-cardiovascular causes [1]. Indeed, previous studies showed the detrimental effect of multimorbidity (i.e., the presence of concurrent comorbidities, beyond AF) and, more generally, of the increasingly complex clinical phenotypes on the prognosis of the general population of patients with AF [3]. As such, international guidelines now put increasing emphasis on the need for integrated management of AF, with care pathways specifically focusing on the comprehensive and active management of comorbidities and associated risk factors [4,5].

In this issue of IJC Heart & Vasculature, van Deutekom, Velt and colleagues present a post-hoc analysis of the RACE II trial (a randomized controlled trial of strict vs. lenient control in 614 patients with recent-onset permanent AF), aimed at investigating the prevalence of multimorbidity, and its associated outcomes [6]. The authors considered nine different comorbidities (including non-cardiovascular ones, such as diabetes mellitus, obesity, renal dysfunction, and respiratory disease), and categorized patients as having 0–1, 2–3, or ≥4 comorbidities; patients with 2 or more comorbidities accounted for 65 % of the total cohort. After 3 years of follow-up, patients with ≥4 comorbidities had approximately 3-fold higher risk of a primary composite outcome of cardiovascular death, stroke/systemic embolism, and other non-fatal cardiovascular outcomes. Rates of all-cause death increased with the number of comorbidities (3.3 %, 5.4 % and 12.5 % for 0–1, 2–3 and ≥4 comorbidities, respectively), and non-cardiovascular deaths were similarly higher in patients with ≥4 comorbidities. Of note, the lack of statistical significance for some of the comparisons should be interpreted with caution, considering the limited power to detect differences in some subgroups.

The authors of this study should be praised for investigating one of the most important aspects of contemporary management of AF patients, and their findings appear valuable. Beyond confirming the detrimental effect of multimorbidity on the prognosis of patients with AF, the study also reaffirms the dose–response relationship of incremental number of comorbidities with the risk of cardiovascular outcomes and mortality, similar to what has been found in previous studies [3,7]. More generally, these findings expand previous observations from the RACE V study, in which the same authors showed that multimorbidity (and each additional comorbidity) increases the risk of AF progression from paroxysmal to more sustained form, being also associated with symptoms severity [8]. From a clinical standpoint, the increase in the risk of non-cardiovascular mortality appears particularly important, especially when considering the epidemiological evidence pointing to it as a potential unmet need in complex patients with AF [1]. In this context – and notwithstanding the increased risk of cardiovascular mortality, which still accounted for the majority of deaths in patients with multimorbidity − the results of this post-hoc analysis of the RACE II trial remark the risk of patients with a high burden of comorbidities and complex clinical profiles, as were those with ≥4 comorbidities.

In the contemporary epidemiological setting, the results of this study have important clinical implications, and should prompt some reflections. First, the focus on the cumulative number of comorbidities is pragmatic and gives insights on the dose–response relationship of multimorbidity with adverse outcomes. Nonetheless, a deeper understanding of how multimorbidity and clinical complexity unfold in patients with AF would also require focusing on the *phenotype* of multimorbidity. Indeed, comorbidities do not occur isolated or at random, and their combination often follow identifiable patterns, and also lead to polypharmacy. Phenotypes of multimorbidity and polypharmacy influence treatments and prognosis of patients with AF [7,9,10], and their identification and recognition can be useful to tailor more directed and specific strategies to improve outcomes. Second, authors were able to consider a limited number of non-cardiovascular comorbidities, due to data availability; however, the impact of other diseases (e.g. dementia, liver and musculoskeletal diseases, depression and other psychiatric comorbidities) is likely important, albeit still poorly investigated in patients with AF. Therefore, a broader investigation and more granular reporting of non-cardiovascular comorbidities and risk factors is needed – both in clinical practice and in research studies − to inform our knowledge on the impact of these diseases on the prognosis of patients with AF.

Finally, the findings of this study confirm that AF patients with more complex clinical profiles (such as those with multimorbidity and polypharmacy) require a holistic, multidisciplinary and comprehensive clinical approach. The observation that >98 % of patients in the RACE II trial were anticoagulated suggests that while optimal stroke prevention remains the cornerstone of AF management, further strategies are needed to counteract the detrimental effect of multimorbidity and clinical complexity in patients with AF.

In this scenario, the ‘Atrial fibrillation Better Care’ (ABC) pathway was proposed in 2017 to implement an integrated approach to the management of patients with AF [11], and has since been associated with improved prognosis in two independent randomized trials [12,13] and many observational cohort studies, including in subgroup of patients with multimorbidity or who are clinically complex [10,14,15]. Similar approaches are now recommended by international guidelines to manage patients with AF [4,5].

Ultimately, the study from van Deutekom, Velt and colleagues reinforces the need to account for the increasing complexity of patients with AF, which requires appropriate response from healthcare systems. Optimal management of this complexity necessarily requires large-scale implementation of integrated care approaches – particularly in those individuals with a high burden of comorbidities and complex clinical profiles, who have the highest absolute risk of adverse outcomes and mortality, and for whom dedicated efforts to address their complex health needs are needed to improve prognosis.

## Declaration of competing interest

The authors declare the following financial interests/personal relationships which may be considered as potential competing interests: GFR reports consultancy for Boehringer Ingelheim and an educational grant from Anthos. No fees were directly received personally. GYHL has been consultant and speaker for BMS/Pfizer, Boehringer Ingelheim, Anthos and Daiichi-Sankyo. No fees are directly received personally. All the disclosures happened outside the submitted work. He is a National Institute for Health and Care Research (NIHR) Senior Investigator and co-PI of the AFFIRMO project on multimorbidity in AF (grant agreement No 899871), TARGET project on digital twins for personalised management of atrial fibrillation and stroke (grant agreement No 101136244) and ARISTOTELES project on artificial intelligence for management of chronic long term conditions (grant agreement No 101080189), which are all funded by the EU’s Horizon Europe Research & Innovation programme.
